# Impact of prior knee surgery on change in knee pain, quality of life, and walking speed following supervised education and exercise therapy: an analysis of 30,545 people with knee osteoarthritis

**DOI:** 10.1007/s10067-024-07195-w

**Published:** 2024-10-28

**Authors:** Dorte T. Grønne, Dilara M. Sari, Søren T. Skou, Ewa M. Roos, Ilksan Demirbüken, Jonas B. Thorlund

**Affiliations:** 1 Research Unit for Musculoskeletal Function and Physiotherapy, Department of Sports Science and Clinical Biomechanics, University of Southern Denmark, Odense, Denmark; 2 The Research and Implementation Unit PROgrez, Department of Physiotherapy and Occupational Therapy, Næstved-Slagelse-Ringsted Hospitals, Slagelse, Denmark; 3 Department of Physiotherapy and Rehabilitation, Marmara University, Istanbul, Turkey; 4 Research Unit for General Practice, Department of Public Health, University of Southern Denmark, Odense, Denmark

**Keywords:** Exercise therapy, Knee osteoarthritis, Knee pain, Prior surgery

## Abstract

To investigate the impact of prior knee surgery on changes in outcomes following an 8-week supervised patient education and exercise therapy program in patients with knee osteoarthritis. Patients were classified according to knee surgery in the most affected knee joint (yes/no) prior to enrolment in the Good Life with osteoArthritis in Denmark (GLA:D^®^) program. Between-group differences in outcome changes from baseline to 3 months follow-up were evaluated using linear regression stratified by sex. Outcomes were knee pain intensity (VAS, 0–100 mm), joint related quality of life (Knee Injury and Osteoarthritis Outcome Score Quality of Life subscale score (KOOS QOL, 0–100)) and walking speed (40-m fast-paced walk test). To evaluate clinically relevant between-group differences, proportions of patients reaching a threshold of minimal important change in the surgery and non-surgery groups were compared. Among 30,545 patients, 27% (*n*, 8254) had prior surgery in the most affected knee. The prior surgery and the non-surgery group experienced improvements in all outcomes with minor between-group differences in change in pain intensity (males, 0.03 95% CI − 0.9 to 1.0; females, 1.3 95% CI 0.6 to 2.1); KOOS QOL (males, 0.3 95% CI − 0.4 to 0.9; females 0.02 95% CI − 0.5 to 0.5); and walking speed (males, 0.01 95% CI − 0.01 to 0.02; females 0.01 95% CI 0.003 to 0.02). The responder analysis showed no clinically relevant between-group differences in improvements. Previous knee surgery does not seem to modify the clinical outcome following exercise therapy and patient education in patients with knee osteoarthritis.

**Supplementary Information:**

The online version contains supplementary material available at 10.1007/s10067-024-07195-w.

**Table:** 

**Key Points**
• *About one in four patients with knee osteoarthritis participating in an 8-week supervised patient education and exercise therapy program had previous knee joint surgery in the most affected knee.* • *Those with prior knee surgery were more likely to be males, to have prior knee injury, to have a longer symptom duration, and to have a high physical activity level compared to those without previous knee surgery.* • *Patients with prior knee surgery did not experience clinically relevant less change in knee pain, joint related quality of life, and walking speed after participation in the intervention, compared to those without previous knee surgery.* • *Patients with previous knee surgery can expect clinical outcomes following exercise therapy and patient education similar to patients without prior knee joint surgery.*

## Introduction

Previous knee surgery is a major risk factor for knee osteoarthritis (OA) [1], and this group of patients may represent a distinct subgroup of knee OA patients [2]. Even years after surgery, there may still be deficits in muscle strength, which is associated with impaired knee function and lower limb proprioception as well as knee OA [3–6]. International guidelines recommend a multimodal, individualized non-surgical treatment approach as the first-line treatment for patients with knee OA; the core treatment being exercise, education, and, if indicated, weight management [7, 8]. Yet, it is currently unclear if the outcome after patient education and supervised exercise treatment differs for patients previously having had knee surgery compared to patients without previous surgery. Information about subgroups of patients with a particular good or bad treatment outcome is important for both patients and clinicians when discussing and balancing treatment expectations and can potentially provide information for tailored care. Therefore, the aim of this study was to investigate if previous knee surgery was associated with poorer outcomes in knee pain, joint related quality of life, and walking speed after participation in an 8-week combined patient education and supervised exercise therapy program in patients with knee OA.

## Materials and methods

### Design

In this registry-based cohort study, we used data from the Good Life with osteoArthritis in Denmark (GLA:D^®^) registry. GLA:D^®^ is a nationwide treatment program with the aim of supporting implementation of knee and hip OA guidelines into clinical practice. The GLA:D^®^ treatment program consists of 2–3 sessions of supervised patient education and 12 sessions of group-based neuromuscular exercise (60 min. per session, twice weekly). The exercise therapy program is based on a neuromuscular exercise program for OA [9, 10] and is delivered by certified physiotherapists. The GLA:D^®^ registry contains a mix of objectively measured, therapist-reported, and patient-reported outcomes. A detailed description of the GLA:D^®^ program has previously been published [11].

### Ethics

This study complies with the Strengthening the Reporting of Observational Studies in Epidemiology (STROBE) statement for reporting of the observational studies [12]. Patient consent was not required according to the Danish Data Protection Act, as personal data were processed exclusively for research and statistical purposes. The GLA:D^®^ registry has been approved by the Danish Data Protection Agency (SDU; 10.084). According to the local ethics committee of the North Denmark Region, ethics approval of GLA:D^®^ is not needed.

### Study population

Patients with knee OA enrolled in the GLA:D^®^ program between January 2013 and August 2023 with available baseline information about previous knee joint surgery in the most affected joint were included in the current study and patients who did not have complete information on the primary outcome (i.e., knee pain intensity) were excluded from the analysis. Also, patients who reported surgery in any knee or hip joint between baseline and 3 months follow-up were excluded from the analysis as this potentially could influence the outcome. As radiographs are not needed for a clinical diagnosis of OA [13], the inclusion criteria for GLA:D^®^ was a clinical diagnosis of knee or hip OA evaluated by the treating physiotherapists but only patients with knee OA as their primary complaint were included in the current study. At a 2-day course provided by the University of Southern Denmark, all therapists were certified to deliver the intervention and to provide a clinical diagnosis of knee OA and rule out other reasons for the symptoms than OA. Using a clinical diagnosis of Knee OA increases the possibility of an early diagnosis, which is optimal when providing this first-line treatment. Nevertheless, 86% of participants in GLA:D^®^ report to have had a radiograph of their knee joint taken and thereof 92% report that it showed signs of knee OA [14]. The exclusion criteria in GLA:D^®^ are as follows: another reason than OA for the joint symptoms (e.g., tumor, inflammatory joint disease, or sequelae after hip fracture); other symptoms that are more pronounced than the OA symptoms (e.g., chronic generalized pain or fibromyalgia) or inability to understand Danish [11].

### Exposure

For the main analysis, patients were classified into two groups according to whether they had a previous surgery in the most affected knee joint or not (yes/no). This information was self-reported to the physiotherapist at the baseline visit. The self-reported information on previous joint surgeries has previously been validated against registry-based data showing a moderate agreement between self-report and registry-based information [15]. In a secondary analysis, patients were categorized according to the type of surgery they underwent, if this information was available (the information was only collected from May 2016 to March 2022, Supplementary Appendix, Fig. S1). Due to low numbers in certain surgery categories, the types of surgeries were classified into three groups: (1) knee replacement surgery, (2) reconstruction of the anterior and/or posterior cruciate ligaments (ACL/PCL), and (3) arthroscopy (i.e., excluding ACL/PCL reconstruction but including surgery to the meniscus, removal of loose bodies, synovectomy, debridement, microfracture, and diagnostic arthroscopy). As some patients had more than one previous surgical procedure, they were classified according to the most invasive procedure according to the order they are listed, i.e., “knee replacement surgery” considered most invasive and “arthroscopy” considered least invasive. Patients with other surgeries (e.g., collateral ligament reconstruction, patellae surgery, osteotomy, and unknown surgery) were not included in the sub-analysis as the category consisted of a variety of different surgeries. In a sensitivity analysis, patients were categorized according to whether they reported previous surgery in any of the four knee/hip joints or not (yes/no) when this information was available (only collected from August 2017 and onwards).

### Outcomes

Information on outcomes were prospectively collected at baseline (prior to the intervention) and after the intervention (approximately at 3 months). The main outcome was a change in knee pain intensity in the most affected joint during the last month/week from baseline to 3 months. The timespan that the question referred to was changed from “last month” to “last week” in June 2020 and an unpublished analysis from the GLA:D^®^ registry showed no differences in mean scores after the change. Knee pain intensity was evaluated on a 0–100 mm Visual Analog Scale (VAS) ranging from “no pain” to “worst pain.” This way of assessing pain intensity has been widely used in diverse adult populations, including those with rheumatic diseases, and has good test–retest reliability and construct validity in this patient group [16]. Secondary outcomes were change in joint related quality of life and walking speed from baseline to after the intervention. Joint related quality of life was assessed with the Knee Injury and Osteoarthritis Outcome Score Quality of Life subscale (KOOS QOL), (0–100 (worst to best)). The KOOS QOL is widely used in patients with OA and has good psychometric properties [17–19]. Walking speed was assessed with the 40-m fast-paced walk test measuring how long time it takes the patient to walk 40 m on a 4 × 10 m track. The patient conducted the test in the clinic under instruction from the treating physiotherapist, and the test results were recalculated to meters per second (m/s). The test is recommended by the Osteoarthritis Research Society International as a core outcome measure to assess physical function in people with knee OA [20] and has good psychometric properties [21]. A difference of a minimum 15 mm change in pain intensity [22], 10 points in KOOS QOL score [17], and 0.095 m/s in walking speed [23] were considered minimal important changes (MICs).

### Statistical analysis

Patient characteristics were presented as mean with standard deviation (SD), median with interquartile range (IQR), or as frequencies and percentages as appropriate for the total population and stratified according to previous knee surgery status. The impact of previous knee surgery on change in knee pain intensity, joint related quality of life, and walking speed was evaluated as the between-group differences in outcome change scores in patients with or without previous knee surgery in the most affected joint using a linear regression model stratified by sex. Sex was the only covariate that was taken into account in the analyses as other potentially relevant information in the GLA:D^®^ registry was collected at baseline, i.e., violating the temporal assumption as the patients underwent surgery prior to baseline. To support the evaluation of whether the between-group differences were clinically relevant or not, we conducted a responder analysis where the proportion of patients in the prior surgery/non-surgery groups reaching MIC thresholds were calculated and compared. In lack of consensus-based thresholds, an arbitrary absolute difference of 20% was a priory defined as between-group differences to be considered clinically relevant. In a subgroup of patients with available information on the type of surgery, a linear regression analysis evaluating the between-group difference in change in knee pain intensity according to type of surgery was conducted. As the independent variable in the regression analyses was categorical, dummy variables were created and included in the regression models with the “no surgery” group as reference group. The potential influence of surgery/surgeries in other knee/hip joints than the index knee joint was investigated in a sensitivity analysis where the outcome analysis was repeated stratified to whether the patient had prior surgery in any knee/hip joint or not in a subgroup of patients where this information was available. To ensure that the rather long inclusion time (i.e., 10 years) did not influence the results, we conducted a sensitivity analysis repeating the main analysis to patients enrolled during the last 5 years. The precision of the estimates was assessed with 95% confidence intervals and all model assumptions were met. All analyses were conducted using SAS V.9.4 (SAS Institute, NC, USA).

## Results

**Fig. 1:**
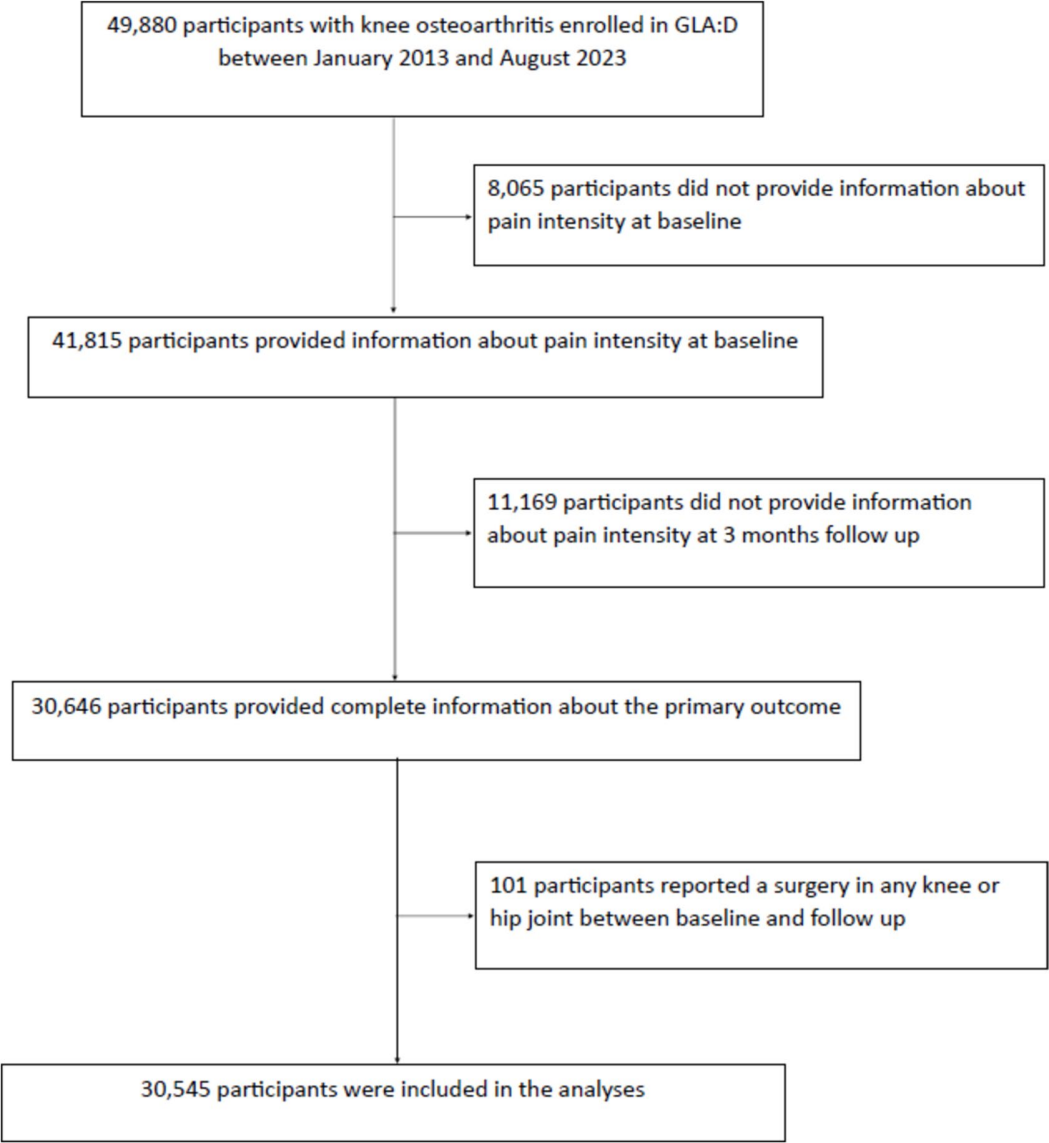
Flow diagram

A total of 30,545 patients with knee OA were included in the study (Fig. 1). The mean age was 65 years, 70% were females, and the mean BMI was 29 kg/m^2^. In total, 27% (*n*, 8254) reported prior knee surgery in the most affected joint (Table 1). Among those with prior knee surgery in the most affected knee, more were males (37% vs. 27%), more had prior knee injury (87% vs. 38%), the median symptom duration was higher (24 months vs. 12 months), and more had a high physical activity level (34% vs. 28%) compared to those without previous knee surgery in the index knee (Table 1). The non-included participants were similar to the included participants according to the available measures (Supplementary Appendix, Table S1).

**Table 1:** Baseline characteristics of the patients included in the analysis

	Total (*n*, 30,545)	Prior surgery in index knee joint (*n*, 8254)	No prior surgery in index knee joint (*n*, 22,291)
Age (years), mean (SD)	65.2 (9.4)	62.7 (9.6)	66.2 (9.2)
Sex (female), % (*n*)	70.2 (21,436)	62.6 (5168)	73.0 (16,268)
BMI (kg/m^2^), mean (SD)	28.8 (5.4)	28.5 (5.0)	28.9 (5.5)
Educational level^a^ (low), % (*n*)	15.3 (4674)	13.2 (1087)	16.1 (3587)
Bilateral symptoms^b^, % (*n*)	45.1 (13,769)	45.8 (3783)	44.8 (9986)
Knee joint instability^c^, % (*n*)	52.9 (6557)	58.9 (1789)	51.0 (4768)
Previous injury of the knee, % (*n*)	51.0 (14,813)	86.5 (6700)	38.1 (8113)
Joint symptoms duration^d^ (months), median (IQR)	12 (6–48)	24 (8–72)	12 (6–36)
Waitlisted for surgery of the knee, % (*n*)	1.6 (473)	2.3 (192)	1.3 (281)
Number of bodily pain areas (0–56), % (*n*)			
0–1	10.6 (3119)	10.0 (786)	10.8 (2333)
2	39.2 (11,543)	38.2 (3018)	39.6 (8525)
3–4	27.1 (7975)	27.9 (2199)	26.8 (5776)
≥ 5	23.1 (6814)	24.0 (1891)	22.8 (4923)
Number of comorbidities^e^, % (*n*)			
0	36.1 (10,639)	39.6 (3120)	34.8 (7519)
1	37.4 (11,018)	36.1 (2847)	37.8 (8171)
2	17.7 (5215)	16.1 (1267)	18.3 (3948)
≥ 3	8.9 (2628)	8.2 (648)	9.2 (1980)
UCLA activity level^f^ mean (SD)			
Low	31.2 (9524)	29.5 (2433)	31.8 (7091)
Moderate	38.9 (11,881)	36.5 (3012)	39.8 (8869)
High	299 (9126)	34.0 (2805)	28.4 (6321)
Pain medication use (yes), % (*n*)			
Overall	61.6 (18,818)	62.8 (5187)	61.2 (13,996)
Paracetamol	52.5 (16,039)	52.3 (4316)	52.7 (12,047)
NSAIDs	34.7 (10,598)	37.9 (3131)	33.4 (7649)
Opioids	5.4 (1663)	6.6 (544)	5.1 (1162)
Prior surgeries in any knee/hip, % (*n*)			
No	62.5 (11,257)	0.0 (0)	83.3 (11,257)
Index knee	25.0 (4508)	100.0 (4508)	0.0 (0)
Other knee	15.6 (2811)	27.90 (1218)	11.8 (1593)
Hip	5.0 (909)	4.0 (182)	5.4 (727)
Types of prior surgery in index knee, % (*n*)	n/a		n/a
Knee replacement surgery		2.1 (109)	
ACL/PCL reconstruction		6.0 (317)	
Arthroscopy		81.6 (4318)	
Other surgeries		10.4 (551)	
Pain intensity (VAS 0–100), mean (SD)	46.8 (22.0)	48.0 (22)	46.3 (22)
KOOS-12 pain (0–100), mean (SD)	50.7 (15.5)	49.8 (15.5)	51.0 (15.5)
KOOS-12 function (0–100), mean (SD)	57.9 (18.3)	56.5 (18.2)	58.3 (18.3)
KOOS-12 QOL (0–100), mean (SD)	45.7 (15.1)	42.7 (14.7)	46.8 (15.1)
KOOS-12 summary score (0–100), mean (SD)	51.5 (14.3)	49.7 (14.1)	52.1 (14.3)
Walking speed^g^ (m/sec), mean (SD)	1.49 (0.33)	1.55 (0.33)	1.47 (0.33)

*ACL/PCL* anterior and/or posterior cruciate ligaments, *BMI* body mass index, *IQR* interquartile range, *KOOS-12* knee injury and osteoarthritis outcome 12-item short form, *n* number, *NSAID* non-steroidal anti-inflammatory drugs, *SD* standard deviation, *UCLA* University of California, Los Angeles Activity score, *VAS* Visual Analog Scale. Missing values due to not collected in the whole time period: Knee joint instability, 18,151; previous knee joint injury, 1498; symptom duration, 2358; number of bodily pain areas, 1094; number of comorbidities, 1045; prior surgeries in any knee/hip, 10,660; types of surgeries, 2959; KOOS-12 pain subscale, 11.011; KOOS-12 function subscale, 11.011; KOOS-12 summary score, 11.011. Missing values due to non-response: BMI, 149; educational level, 10; walking speed, 1742; KOOS-12 QOL, 57; UCLA activity scale, 14. ^a^Educational level: low (no education completed or primary school is highest level completed). ^b^Bilateral symptoms include pain and/or functional limitations associated with knee osteoarthritis from both knee joints. ^c^Knee joint instability was self-reported answering the question 'Have you felt your knee give away or let you down within the last week?' categorized into 'No' (Never/Rarely) or 'Yes' (Sometimes/Most of the time/All the time). ^d^Symptom duration include time (months) since onset of pain or functional limitations associated with knee osteoarthritis. ^e^Count from a list of self-reported comorbidities: Hypertension, cardiovascular diseases, lung diseases, diabetes, stomach diseases, kidney/liver diseases, blood diseases, cancer, depression, rheumatoid arthritis, neurological disorders, other medical diseases. ^f^UCLA-University of California, Los Angeles activity scale: low (1–4), moderate (5–6), high (7–10). ^g^Walking speed assessed with the 40 m fast passed walking test

Mean improvements were observed from baseline to 3 months follow up in all outcomes both in those with and without prior knee surgery in the index knee (Table 2). We observed a statistically significant between-group difference in change in knee pain in females in favor of the no surgery group but not in males (between-group differences, males, 0.03 mm, 95% CI − 0.9 to 1.0; females, 1.3 mm 95% CI 0.6 to 2.1) (Table 2). The responder analysis showed that there were no clinically relevant between-group differences in proportions of patients reaching the MIC for any of the outcome scores (Table 2).

**Table 2:** Outcome scores at baseline, 3 months follow-up, change between baseline and follow-up, difference in outcome score change between prior surgery groups and percentages of responders, stratified by sex

		Baseline Mean (95% CI)	Follow-up Mean (95% CI)	Mean change baseline to 3 m follow-up (95% CI)	Between group difference in mean change (95% CI)	Responders % (*n*)
Knee pain intensity (VAS, 0–100 mm)^a^						
Males	No surgery in index knee	42.3 (41.8; 42.8)	32.3 (31.8; 32.9)	− 10.0 (− 10.5; − 9.4)	Reference	38.0 (2290)
	Prior surgery in index knee	44.9 (44.2; 45.7)	35.0 (34.2; 35.8)	− 9.9 (− 10.7; − 9.1)	0.03 (− 0.9; 1.0)	38.2 (1178)
Females	No surgery in index knee	47.8 (47.4; 48.1)	33.6 (33.3; 33.9)	− 14.2 (− 14.5; − 13.8)	Reference	46.7 (7595)
	Prior surgery in index knee	49.8 (49.2; 50.4)	37.0 (36.4; 37.6)	− 12.8 (− 13.5; − 12.2)	1.3* (0.6; 2.1)	44.0 (2276)
Joint related quality of life (KOOS QOL, 0–100)^b^						
Males	No surgery in index knee	48.4 (48.0; 48.8)	53.5 (53.0; 53.9)	5.0 (4.6; 5.4)	Reference	35.9 (2108)
	Prior surgery in index knee	44.5 (43.9; 45.0)	49.8 (49.2; 50.4)	5.3 (4.8; 5.8)	0.3 (− 0.4; 0.9)	35.5 (1095)
Females	No surgery in index knee	46.2 (46.0; 46.5)	52.8 (52.5; 53.0)	6.5 (6.3; 6.8)	Reference	39.1 (6359)
	Prior surgery in index knee	41.7 (41.3; 42.1)	48.2 (47.8; 48.7)	6.5 (6.2; 6.9)	0.02 (− 0.5; 0.5)	39.1 (2022)
Walking speed (40 m FPWT, m/sec)^c^						
Males	No surgery in index knee	1.54 (1.53; 1.54)	1.67 (1.66; 1.68)	0.13 (0.12; 0.13)	Reference	54.7 (2294)
	Prior surgery in index knee	1.61 (1.59; 1.62)	1.74 (1.73; 1.76)	0.13 (0.12; 0.14)	0.01 (− 0.01; 0.02)	55.5 (1208)
Females	No surgery in index knee	1.45 (1.44; 1.45)	1.57 (1.56; 1.58)	0.13 (0.12; 0.13)	Reference	54.9 (6336)
	Prior surgery in index knee	1.50 (1.50; 1.51)	1.64 (1.63; 1.65)	0.14 (0.13; 0.14)	0.01* (0.003; 0.02)	56.2 (2062)

*40 m FPWT* 40 m fast-paced walk test, *CI* confidence interval, *KOOS QOL* Knee Injury and Osteoarthritis Outcome Score Quality of Life subscale score, *n* number, *SD* standard deviation, *VAS* Visual Analog Scale. ^a^*n*, 30,545; ^b^*n*, 30,536; ^c^*n*, 21,588

^*^Statistically significant between-group difference

In the subgroup analysis evaluating the impact of type of surgery on patient outcome, 19,885 participants were included (Supplementary Appendix, Table S2). Females with prior knee joint replacement surgery or knee joint arthroscopy in the index knee had a statistically significant smaller pain reduction compared to those without prior knee surgery, but according to the responder analysis, the between-group differences were not considered clinically relevant (Table 3). The results from the sensitivity analysis (*n*, 18,019) evaluating the impact of prior surgery in any of the four joints on outcome measures were similar to the results from the main analysis (Supplementary Appendix, Table S3). Also, restricting the analysis to patients enrolled during the last 5 years showed similar results (not shown).

**Table 3:** Outcome scores at baseline, 3 months follow-up, change between baseline and follow-up, difference in outcome score change between prior surgery groups including type of surgeries and percentages of responders, stratified by sex

		Baseline Mean (95% CI)	Follow-up Mean (95% CI)	Mean change baseline to 3 m follow-up (95% CI)	Difference in change (95% CI)	Responders % (*n*)
Knee pain intensity (VAS, 0–100 mm)						
Males	No surgery in index knee^a^	42.4 (41.7; 43.0)	32.6 (31.9; 33.3)	− 9.8 (− 10.4; − 9.1)	Reference	37.8 (1595)
	Knee replacement surgery^b^	37.2 (30.1; 44.3)	34.6 (26.2; 42.9)	− 2.7 (− 12.4; 7.0)	7.1 (− 0.9; 15.1)	30.0 (9)
	ACL/PCL reconstruction^c^	44.1 (40.1; 48.1)	33.2 (29.7; 36.7)	− 10.9 (− 14.2; − 7.6)	− 1.1 (− 4.8; 2.6)	41.8 (59)
	Arthroscopy^d^	45.1 (44.0; 46.1)	35.3 (34.2; 36.3)	− 9.8 (− 10.9; − 8.7)	− 0.03 (− 1.3; 1.2)	38.2 (616)
Females	No surgery in index knee^e^	47.9 (47.4; 48.3)	33.7 (33.2; 34.1)	− 14.2 (− 14.6; − 13.8)	Reference	46.8 (5113)
	Knee replacement surgery^f^	42.6 (37.8; 47.4)	36.0 (30.8; 41.3)	− 6.6 (− 11.5; − 1.7)	7.6* (2.4; 12.9)	34.2 (27)
	ACL/PCL reconstruction^g^	46.5 (43.3; 49.8)	33.4 (30.2; 36.6)	− 13.1 (− 16.8; − 9.4)	1.1 (− 2.4; 4.6)	41.6 (73)
	Arthroscopy^h^	50.0 (49.1; 50.8)	37.2 (36.4; 38.1)	− 12.7 (− 13.6; − 11.8)	1.5* (0.5; 2.5)	44.4 (1201)

*ACL/PCL* anterior and/or posterior cruciate ligaments, *CI* confidence interval, *n* number, *SD* standard deviation, *VAS* Visual Analog Scale. ^a^*n*, 4220; ^b^*n* 30; ^c^*n* 141; ^d^*n* 1613; ^e^*n*, 10,921; ^f^*n*, 79; ^g^*n*, 176; ^h^*n*, 2705. *Statistically significant between-group difference

## Discussion

We found that about one in four patients with knee OA enrolled in a supervised patient education and exercise therapy program (GLA:D^®^) had previous surgery in the most affected knee joint. Patients with prior knee surgery did not experience clinically relevant inferior change in knee pain, joint related quality of life, and walking speed after participation in the intervention, compared to those without previous knee surgery.

One in four patients who was enrolled in the patient education and exercise therapy program previously had knee surgery, which is in line with results among patients participating in the Swedish “Better management of patients with OsteoArthritis” (BOA) program [24]. Also, we found that those with prior knee surgery were more likely to be male, have a history of previous knee injury, experience longer duration of joint symptoms, and were more likely to have a high physical activity level. These findings correspond to the elevated risk of knee OA in people with prior knee injury, as a knee injury is a typical reason for knee surgery [1], and that incidence of knee injuries is higher among males, particularly due to their greater involvement in sports activities [2]. Paradoxically, knee OA patients with prior knee injury tend to have more pronounced OA symptoms, longer symptom duration, and general better health status [2], and in the current study, we confirmed that this also applies to those with prior knee surgery. Nevertheless, we found that those with prior knee surgery did not differ from those without prior surgery in terms of treatment outcomes from the GLA:D^®^ program.

Although we did observe a statistically significant between-group difference in change in knee pain in females in favor of the no surgery group, it was only small and not supported by evaluations of the other outcomes or in males, nor considered clinically relevant. As such, previous knee surgery did not influence patient outcomes, which is consistent with the results from a study evaluating factors associated with the outcome in a similar intervention delivered in the BOA program in Sweden, where they found a statistically significant but small association between previous surgery and pain reduction at 3 months in favor of those without previous surgery [24]. Notably, the association was stronger when evaluating the pain reduction 12 months after baseline, which was not evaluated in the current study. Extending previous research, our study evaluated the influence of type of surgery as well as the impact of a surgery in any knee/hip joint stratified by sex but did not observe any clinically important between-group differences. Previously, a range of different factors has been studied as potential moderators of the effect of exercise therapy targeting people with knee OA, but in general, there is a lack of ability to identify factors that significantly modify the effect [25] which was also the case for prior surgery in the current study.

The target group for the GLA:D^®^ program is people with knee or hip osteoarthritis, thus typically not patients with previous joint replacement surgery in the joint for which they seek care. However, joint replacement surgery is not an explicit exclusion criterion for enrollment, and therefore, a small proportion of GLA:D^®^ participants (0.5%) have had a previous joint replacement surgery in the most affected joint. Around 15 to 20% develop chronic pain after a joint replacement surgery [26] which might explain their participation in the GLA:D^®^ program. In the current study, those with a previous knee joint replacement surgery in the most affected joint experience a lower degree of pain relief, compared to those without previous surgery even though the confidence intervals are wide and the magnitude of the between-group difference in patients reaching the MIC did not exceed the a priori defined threshold for clinical relevance. People with chronic pain after prior joint replacement surgery might have an inferior pain relief compared to those without previous surgery, which may be due to that exercise therapy does not seem to have an additional effect on chronic pain in addition to patient education in this population [27].

We showed that a large part of the population engaging in a patient education and exercise therapy program targeting knee OA symptoms had a previous knee surgery. Thus, the results from our study are valuable for future healthcare planning as it suggest that the change in knee pain, joint related quality of life, and walking speed in patients with knee OA after guideline-recommended treatments is not moderated by previous surgery status. This knowledge can be valuable for both patients and clinicians when discussing and managing treatment expectations as people with knee osteoarthritis and prior knee surgery can be informed to expect an average outcome from a supervised patient education and exercise therapy program.

The strengths of this study are the large sample size and that we included patients with knee OA from a large clinical cohort, likely with good generalizability to a primary care clinical setting. The information on previous surgery was self-reported and might be prone to recall error and thereby the risk of misclassification; however, the self-reported information on prior surgery has previously been shown to have a moderate agreement with registry-based data, i.e., a perfect agreement between self-report and registry-based data for joint replacement surgery and lower agreement with less invasive knee surgeries [15]. Nevertheless, a risk of misclassification in the current study still exists, potentially underestimating the between-group differences. We were only able to classify patients into few categories according to types of surgeries, which prevented us from analyzing if the results differ according to more detailed information on types of surgeries. Finally, information about time between surgery and enrollment in GLA:D^®^ was not available; thus, we were unable to take this into account in the analyses and interpretation of data. An absolute difference of 20% was a priory defined as between-group differences to be considered clinically relevant. In lack of consensus-based thresholds, this was an arbitrary level that was found to be reasonable; thus, it can be questioned whether an alternative threshold would have been more appropriate [28, 29]. Given the observational nature of the study, it is not possible to ascertain how large a part of the observed changes that are due to the GLA:D^®^ intervention, regression-to-the-mean, or contextual factors. Also due to the temporality of the data collection, we were only able to take sex into account in the analyses, thus residual confounding may occur.

In conclusion, we observed no clinically relevant differences in treatment outcomes in pain, joint related quality of life, and walking speed following a supervised patient education and exercise therapy program regardless of previous knee surgery status. These results suggest that previous knee surgery does not impact the potential benefit of exercise therapy and patient education as effective strategies for managing symptoms and enhancing function in patients with knee OA.

## Supplementary Information

Below is the link to the electronic supplementary material.

Supplementary file1 (PDF 148 KB)

## Data Availability

The data used in this study may be available on reasonable request from the corresponding author. The data are not publicly available due to privacy restrictions.
