# Supplementary material for: Impact of prior knee surgery on change in knee pain, quality of life, and walking speed following supervised education and exercise therapy: an analysis of 30,545 people with knee osteoarthritis
Source: Clin Rheumatol. 2024 Oct 28;43(12):3925–34. doi: 10.1007/s10067-024-07195-w (PMC11582206; doi:10.1007/s10067-024-07195-w)
Supplement: Supplementary file 1 — Supplementary file1 (PDF 148 KB) [file 10067_2024_7195_MOESM1_ESM.pdf]

SUPPLEMENTARY APPENDIX

Clinical Rheumatology

Impact of prior knee surgery on change in knee pain, quality of life, and walking speed following supervised education and exercise therapy: an analysis of 30,545 people with knee osteoarthritis

Dorte T. Grønne, Dilara M. Sari, Søren T. Skou, Ewa M. Roos, Ilksan Demirbüken, Jonas B. Thorlund

Correspondence: Dorte T. Grønne, Department of Sports Science and Clinical Biomechanics, University of Southern Denmark, Campusvej 55, Odense, Denmark; dgronne@health.sdu.dk.

Figure S1. Response options in question about prior knee surgeries

Response options in question about prior knee surgeries collected May 2016 and onwards by the therapist asking the patient (possible to mark several answers)
Diagnostic arthroscopy<br>Arthroscopic meniscal surgery<br>Removal of loose bodies in knee joint<br>ACL reconstruction<br>PCL reconstruction<br>Collateral ligaments reconstruction<br>Debridement<br>Microfracture<br>Surgery for jumper's knee<br>Partial synovectomy<br>Patellae stabilization<br>Osteotomy<br>Arthroplasty<br>Patellae, other<br>Other knee surgeries

Table S1. Baseline characteristics by inclusion status

	Included (n: 30,545)	Not included (n: 19,385)
Age (years), mean (SD)	65.2 (9.4)	65.1 (10.7)
Sex (female), % (n)	70.2 (21,436)	68.7 (13,322)
BMI (kg/m^2), mean (SD)	28.8 (5.4)	29.2 (5.6)
Joint symptoms duration (months), median (IQR)	12 (6-48)	12 (6-36)
Pain medication use (yes), % (n)		
Overall	61.6 (18,818)	63.3 (12,840)
Paracetamol	52.5 (16,039)	54.5 (10,562)
NSAIDs	34.7 (10,598)	33.8 (6,545)
Opioids	5.4 (1,663)	7.0 (1,351)
Prior knee surgery in index knee (yes), % (n)	27.0 (8,254)	26.0 (5,032)
Types of prior surgery, % (n)		
No prior surgery	74.1 (15,414)	74.6 (10,162)
Knee replacement surgery	0.5 (109)	1.0 (131)
ACL/PCL reconstruction	1.6 (317)	1.6 (217)
Arthroscopy	21.1 (4,318)	20.5 (2,788)
Other surgeries	2.7 (551)	2.4 (325)
Walking speed[c] (m/sec), mean (SD)	1.49 (0.33)	1.42 (0.36)

ACL/PCL: Anterior and/or posterior cruciate ligaments, BMI: Body Mass Index, IQR: Interquartile range, n: Number, NSAID: Non-steroidal anti-inflammatory drugs, SD: Standard deviation. Missing values due to not collected in the whole time period: Symptom duration: 3,533, Types of surgeries: 15,871. Missing values due to non-response: BMI: 331; Walking speed: 3,472.

Table S2. Type of surgeries stratified by sex

	Total (n: 20,436)	Males (n: 6,239)	Females (n: 14,197)
Types of prior surgery in index knee, % (n)			
No prior surgery	74.1 (15,141)	67.6 (4,220)	76.9 (10,921)
Knee replacement surgery	0.5 (109)	0.5 (30)	0.6 (79)
ACL/PCL reconstruction	1.6 (317)	2.3 (141)	1.2 (176)
Arthroscopy	21.1 (4,318)	25.9 (1,613)	19.1 (2,705)
Other surgeries	2.7 (551)	3.8 (235)	2.2 (316)

ACL/PCL: Anterior and/or posterior cruciate ligaments, n: number.

Table S3. Outcome scores at baseline, 3 months follow-up, change between baseline and follow-up, and difference in outcome score change between prior surgery in any knee/hip groups, stratified by sex.

		Baseline Mean (95% CI)	Follow-up Mean (95% CI)	Mean change baseline to 3m follow-up (95% CI)	Between group difference in mean change (95% CI)	Responders % (n)
Knee pain intensity (VAS, 0-100 mm) [a]						
Males	No surgery in any knee/hip	41.0 (40.2; 41.8)	30.8 (30.0; 31.5)	-10.2 (-11.0; -9.5)	reference	38.7 (1,194)
	Prior surgery in any knee/hip	44.5 (43.6; 45.3)	35.2 (34.3; 36.1)	-9.3 (-10.2; -8.4)	0.9 (-0.2; 2.1)	37.5 (962)
Females	No surgery in any knee/hip	46.2 (45.7; 46.7)	32.1 (31.6; 32.6)	-14.1 (-14.7; -13.6)	reference	46.5 (3,801)
	Prior surgery in any knee/hip	49.7 (49.1; 50.4)	36.9 (36.2; 37.6)	-12.8 (-13.6; -12.1)	1.3* (0.4; 2.2)	44.6 (1,870)
Joint related quality of life (KOOS QOL, 0-100) [b]						
Males	No surgery in any knee/hip	49.2 (48.6; 49.7)	54.6 (54.0; 55.2)	5.4 (4.9; 6.0)	reference	36.6 (1,129)
	Prior surgery in any knee/hip	44.7 (44.1; 45.3)	50.0 (49.3; 50.7)	5.3 (4.7; 5.9)	-0.1 (-0.9; 0.7)	35.8 (918)
Females	No surgery in any knee/hip	46.8 (46.5; 47.1)	53.9 (53.5; 54.3)	7.1 (6.8; 7.4	reference	40.9 (3,343)
	Prior surgery in any knee/hip	42.2 (41.7; 42.6)	48.5 (47.9; 49.0)	6.3 (5.8; 6.8)	-0.8 (-1.4; 0.2)	38.2 (1,603)
Walking speed (40m FPWT, m/sec) [c]						
Males	No surgery in any knee/hip	1.53 (1.52; 1.55)	1.67 (1.65; 1.68)	0.13 (0.12; 0.14)	reference	55.2 (1,104)
	Prior surgery in any knee/hip	1.58 (1.56; 1.60)	1.71 (1.69; 1.73)	0.13 (0.12; 0.14)	-0.01 (-0.02; 0.01)	54.8 (929)
Females	No surgery in any knee/hip	1.46 (1.45; 1.46)	1.58 (1.57; 1.59)	0.13 (0.12; 0.13)	reference	54.3 (2,939)
	Prior surgery in any knee/hip	1.45 (1.44; 1.46)	1.59 (1.57; 1.60)	0.13 (0.12; 0.14)	0.01 (-0.002; 0.02)	55.3 (1,510)

40m FPWT: 40 meter fast-paced walk test, CI: Confidence interval, KOOS QOL: Knee Injury and Osteoarthritis Outcome Score Quality of Life subscale score, n: Number, SD: Standard deviation, VAS: Visual Analogue Scale. [a] n: 18,019; [b] n: 18,018; [c] n: 11,841.
*Statistically significant between-group difference.
